# Visualizing Rank Deficient Models: A Row Equation Geometry of Rank Deficient Matrices and Constrained-Regression

**DOI:** 10.1371/journal.pone.0038923

**Published:** 2012-06-19

**Authors:** Robert M. O’Brien

**Affiliations:** Department of Sociology, University of Oregon, Eugene, Oregon, United States of America; Queen’s University Belfast, United Kingdom

## Abstract

Situations often arise in which the matrix of independent variables is not of full column rank. That is, there are one or more linear dependencies among the independent variables. This paper covers in detail the situation in which the rank is one less than full column rank and extends this coverage to include cases of even greater rank deficiency. The emphasis is on the row geometry of the solutions based on the normal equations. The author shows geometrically how constrained-regression/generalized-inverses work in this situation to provide a solution in the face of rank deficiency.

## Introduction

The problem of collinear independent variables is well known. When there is collinearity (a linear dependency between independent variables) in a regression model there is no unique solution for the regression coefficients. We say that these regression coefficients are not identified, since there are an infinite number of solutions, rather than a unique set of solutions. To obtain one of these solutions, when the rank deficiency is one, a common strategy is to place one constraint on the regression coefficients (for example, fix two of the regression coefficients to be equal or one of them to be twice as large as another). This typically is accomplished using statistical programs such as [1] or [2] or can be implemented by the use of generalized inverses or by creative recoding the data [3].

This paper does not propose a new method for solving regression problems in the face of collinearity. Instead it offers a general geometric view of the linear dependency problem (collinearity). It shows how the most common approach to solving regression equations in such situations (constrained-regression/generalized-inverses) can be viewed geometrically. Constrained solutions can be implemented by creating specific generalized inverses that incorporate one or more constraints [4], and, importantly, any generalized inverse constrains the solution in both an algebraic and geometric sense. The user of packaged programs will not use this geometry to obtain constrained solutions nor will the researcher use generalized inverses, but this geometry is extremely helpful in understanding: (1) the problem of collinearity, (2) how these constrained solutions work, (3) how many constraints are necessary to identify a solution, and (4) why some constraints do not produce identified solutions. The general geometric perspective may also help in judging what it is that makes a particular constrained solution plausible.

Rank deficient matrices occur when one or more of the independent variables are a linear function of the other independent variables in the model. These sorts of dependencies can occur naturally in the course of research. Four diverse example are: (1) when the total test score consists of the score on the math section plus the score on the verbal section and one wants to assess the independent effects of the total score (TS), the math score (MS), and the verbal score (VS) on college GPA. The linear dependency is VS + MS  =  TS. (2) Separating the effects of educational status (ES), occupational status, (OS) and status inconsistency (SI): SI  =  OS – ES [5]. (3) Disentangling the effects origin status (OrigS), destination status (DS), and the degree of mobility DM: DM  =  DS – OrigS [6]. (6) In demography and epidemiology separating the effects of current age (A), current period (P), and birth cohort (C): C  =  P – A [7] is a vital and important problem [3], [8]–[12]. In these scenarios, each of the independent variables may have an effect on the outcome variable, but in all of these situations the independent variables are linearly dependent.

I expand upon the Age-Period-Cohort model example, because I work directly in this area, and the problem of rank deficiency in this area has generated and continues to generate intense interest in sociology, demography, epidemiology, medicine, and other related areas [3], [8]–[12]. This model comes in two distinct forms. One is simply to code ages in years, cohorts in birth years, and periods in yearly dates. That is, coding all three of these variables as continuous interval-level variables. The second, and most common form, is to code these three variables with dummy variables or effect coding. With dummy variable coding each age-group is coded with a dummy variable except for a reference category, each period is coded with a dummy variable except for a reference category, and each cohort is coded with a dummy variable except for a reference category. Using categorical coding, there are typically many dimensions in the solution space (one for each dummy variable plus the intercept). The model, however, is still rank deficient by one.

The typical solution to this problem is to use constrained regression. Make an assumption about two of the categories such as the effects of the first and second dummy variables for birth cohorts are the same. This will identify the model and produce *a* solution. The problem is that the solutions differ depending upon the constraint imposed and often the solutions differ substantially. Researchers typically use a constrained regression program available in commonly used software programs [1], [2], but these same constrained solutions can be found using matrix algebra by choosing the appropriate generalized inverse [4]. Typically researchers set the constraint based on theory or past research hoping that it is approximately correct [8]. Researchers may also suggest that a particular constraint is the preferred one in general without resorting to substantive theory or research to set the constraint [11]. This author has criticized this approach [10], suggesting that when using constrained regression, constraints should be based, whenever possible, on substantive/theoretical considerations.

In this areas of research facing the problem of structural underidentification, it is helpful to be aware of the geometry of rank deficient models. What does the geometry of rank deficient models look like? How does constrained regression work? Why do some constraints not work? The geometry shows that there are some things we know about all possible solutions when using rank deficient models. For example, in the rank deficient by one situation, the OLS solutions (solutions to the normal equations) all lie on a line in multidimensional space. We can describe this line explicitly: the line is identified. The constraint we use (whether it is implicit or we chose it explicitly) determines one of the points on this line and, thus, one of the infinite number of least squares solutions. Our choice is, of course, subject to error; it is no better than the choice of the constraint used to select that solution. This fact should keep researchers modest in their claims for solutions based on constrained regression.

In each of the four cases of linear dependency discussed above, the matrix of independent variables is one less than full column rank since only two of the independent variables are linearly independent. Adding the third independent variable means that one of the three variables can be determined perfectly from the other two. This three variable model has a rank of two and is rank deficient by one. Because of this linear dependency, no unique solution exists. One way to obtain a solution, however, is to impose a constraint on the possible solutions such as constraining the math test effect on GPA to be half as great as that of the verbal test effect. The constraints are often based on theory or past research. That is, the researcher has some reason to believe that math skills (as measured by the test) should be less important to the overall GPA than verbal skills (as measured by the test). Justifying that the math effect should be one-half as large as the verbal test effect requires precision not often found in social research. Less theoretically, we can obtain a solution by using any appropriate generalized inverse. This identifies the model, but the solutions depend on the constraint employed (generalized inverse used) and different constraints can provide widely divergent results.

Others have written on the geometry of generalized inverses or related topics [13]–[16], but this paper provides a unique, and more intuitive, view. It emphasizes the geometry of the solution space (not the construction of a generalized inverse), it does so from the row perspective (using row equations) rather than a column perspective (using column vectors), and it emphasizes the null space and the hyperspace of solutions that is parallel to the null space. It presents a simpler geometric view of the solutions obtained with generalized-inverses/constrained-regression than these earlier papers; in part, because its scope and purpose are quite different. Our purpose is to provide a simple geometric view of the rank deficiency problem and of how solutions are obtained by using generalized-inverses/constrained-regression when the matrix of independent variables is less than full rank.

## Methods

The method used is straightforward. I begin with simple spaces of one, two, and three dimensions. I then extend this approach to situations with four or more dimensions. Understanding this geometry takes some effort even in the one-, two-, and three-dimensional situations and, obviously, more effort as we move to the geometry of four or more dimensions. To simplify, I will deal throughout with the normal equations associated with Ordinary Least Squares (OLS) regression, since this is the situation most familiar to readers. I begin with the simplest situation, the bivariate case. We subtract the mean of the independent variable from each independent variable scores and the mean of the dependent variable from the dependent variable scores. This leaves us with deviation scores and allows us to consider only the one regression coefficient between these two variables since the intercept is zero. In this situation there is only one normal equation.

In the one independent one dependent variable situation, there are only two quantities needed to find the regression coefficient: the sums of squares for the independent variable (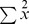) and the sum of products for the independent and dependent variables (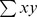). In this two variable situation there is one normal equation
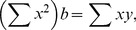
(1)yielding the familiar solution 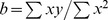. Using matrix algebra, we write this same equation as 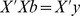. Where *X* is an *n* × 1 vector of the deviations of the scores of the *n* observations on the independent variable and *y* is an *n* × 1 vector of the deviations on the dependent variable for *n* observations. The prime means that the column vector has been transposed (in this case into a row vector). When we carry out the matrix multiplications, we end up with a single equation: equation (1). For concreteness, we create values for 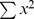 and 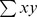, and place them into (1): 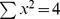 and 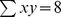. Then we can write (1) as 4*b* = 8 and; thus, *b*  = 2. Geometrically, the solution space has only one dimension (*b*) and equation (1) allows us to solve for a unique point on this line. It determines where on that one-dimension of possible values of *b* the solution lies.

We extend this method by moving to the two independent variable situation. We again center the variables by subtracting their means from them so that all of the variables are in deviation score form. We distinguish between the two independent variables by subscripting them with a one or a two: 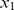 or 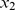. From an algebraic perspective the quantities of interest are 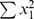, 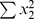, 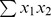, 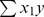, 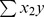. Formulas from introductory texts that cover multiple regression allow one to place these quantities into formulas and solve for the two regression coefficients [17]. The matrix algebra representation remains the same 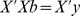, but now the *X* matrix contains two columns (one for each of the independent variables) and *n* rows (one for each of the observations). The vector *b* has two elements one for the regression coefficient for the first independent variable (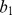) and one for the second independent variable (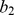). We write out the explicit matrix form of the equations using the sums of squares and cross-products:
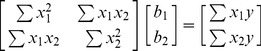
(2)Carrying out the matrix multiplication in (2), we can write the two normal equations:



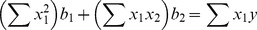


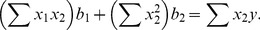
(3)Each of these normal equations is the equation for a line (the general form of the equation for a line: 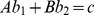). We again supply some appropriate values for the sums of squares and products [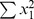, 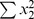, 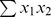, 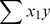, 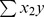] and placing these into (3) produce a set of two normal equations that could result from real data,



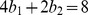


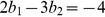
(4)We can solve this two equation system by, for example, substituting 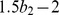 into the first equation for 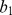, we find that 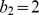 and then knowing 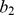 we can easily solve for 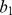
which is equal to 1.

**Figure 1:**
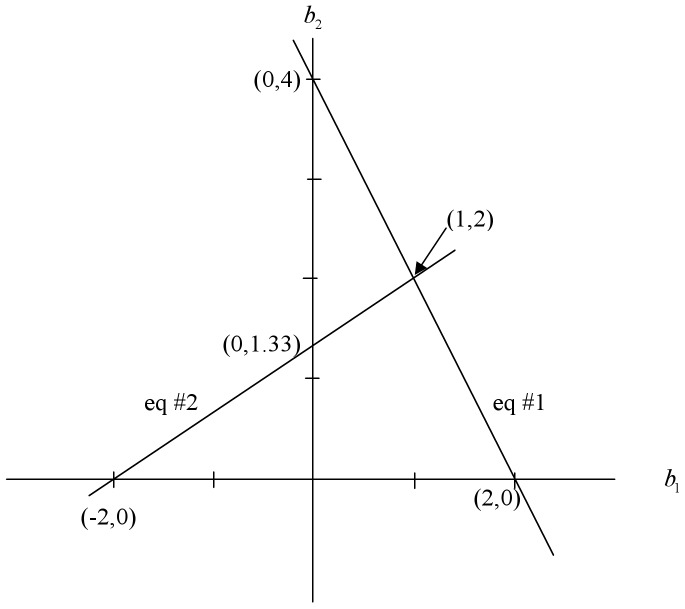
Geometric view of a regression solution in a two-dimensional solution space with no linear dependency. The solution is where the two lines representing equations 1 and 2 intersect (1, 2).

Geometrically, the solution space has two dimensions: one for 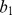 and one for 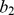. The normal equations in (4) are equations for lines and if these two lines intersect in a point in this two dimensional solution space that point will determine a unique solution to this two equation system. This is depicted in Figure 1. The horizontal axis represents the solutions for 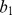
and the vertical line the solutions for 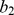. We construct the two lines based on the equations in (4) in the following manner. Using the first equation, if 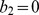 then 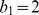 so that one of the points on the line is (2, 0). On the other hand, if 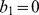 then 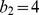 and a second point on this first line is (0, 4) and these two points allow us to draw this first line in the two dimensional solution space. The second line is constructed in the same manner, we set 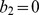 and 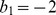, so one point on the line is (−2, 0). If we set 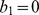 then 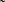, then a second point on the line is (0, 1.33). This allows us to construct the second line. These two lines intersect at (1, 2); that is, 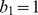 and 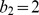. This is the geometric view of the solution to the normal equations with two independent variables. It is likely familiar to most readers (albeit from a different context).

Imagine the situation in which the two equations are linearly dependent, for example:
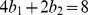


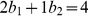
(5)The second equation is one-half times the first equation. There is no unique solution to these equations. When we substitute the second equations value for 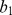 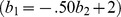 into the first equations value for 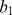 and solve for 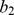, we obtain 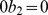, a rather uninformative result since 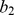 could take on any value. We say that *b*_2_ is not identified. If we substitute the value of 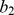 from second equation 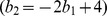 into the first equation, we find that 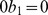. Geometrically we can plot the first equation as before and end up with the line for equation 1 in Figure 1. When we plot the second line, we find that it crosses the 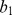
axis at (0, 4) and the 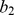 axis at (2, 0). That is, the lines for these two equations coincide. Any solutions to these equations lie on this line. For example, (2, 0) is a solution to both of these equations, as are (0, 4) and (1, 2). There are an infinite number of solutions to these two equations, and they all lie on this line in a space of two dimensions. One informative way to write the equation for this line is as the “vector equation for a line.” That is, as one of the points on the line plus a scalar (*k*) times the “direction of the line”:



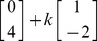
This geometric notion of a line of solutions tells us not only that *b*_1_ and *b*_2_ are not identified; it tells us the combinations of *b*_1_ and *b*_2_ that solve the normal equations. To show how this works, note that we have previously shown that (0, 4) is on the line and it is a solution when *k*  = 0; we have shown that (1, 2) is on this line and it is a solution when *k*  = 1; and we have shown that (2, 0) is on this line and it is a solution when *k*  = 2. Selecting other values for *k* will produce the other points on this line; that is, any of the other solutions to this set of two equations. Importantly, although there are an infinite number of solutions to these two equations, the only solutions are those that lie on this line.

At this point it is appropriate to introduce the null vector. The null vector is the vector that when multiplied times a matrix results in a vector of zeros. We focus on the normal equations and . In this context the null vector is the vector that when premultiplied by 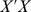
produces a vector of zeros under the condition that not all of the elements of the null vector are zeros. Writing the 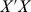
matrix for (5) we have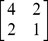 and we note that the vector 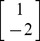
when multiplied time 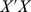 produces the vector 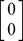. The null vector is 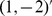, and we represent it as *v*. It is unique up to multiplication by a scalar. There is only one null vector for the 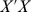 in (5), because there is only one linear dependency (there cannot be more than one linear dependencies with only two independent variables). We note that the line of solutions is parallel to the null vector, since they share the same direction. The null vector is a line running through the (0, 0) point with a slope of minus 2.

The final situation in which it is relatively easy to visualize geometrically the solutions and the problems caused by linear dependencies among the independent variables is the situation in which there are three independent variables. Below is the matrix of sums of squares and cross products in matrix form:
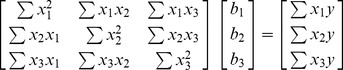
(6)We can write the three normal equations based on this matrix formulation as:



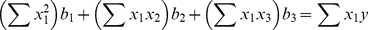


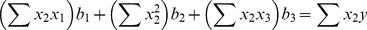
(7)

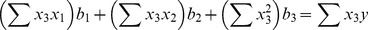



These are the three normal equations that when solved for 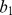, 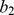, and 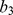 provide the least squares solutions. Geometrically each of these equations represents the equation for a plane: 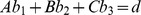. Where *A*, *B*, *C*, and *d* are real numbers. We again can provide some appropriate numbers for these sums of squares and cross-products (in practice, of course, they are derived from observations). This produces the three normal equations for the data:
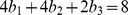


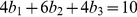
(8)

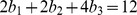
We can solve these equations using substitution as we did to solve the two equation system in (4) or we can use matrix algebra: the solution set is: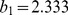, 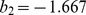, and 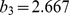. This solution set is the unique least squares solution set for this data.

We can construct our geometric figure as before, except now the solution space has three dimensions one for 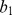, one for 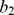, and one for 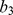. Each of the three equations represents a plane. To construct one of the planes, we can determine where the plane for the first equation crosses the 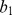 axis; that is, what is the value of 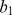 when 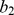 and 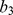 are both equal to zero. The answer is that 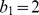; one point on this plane is (2, 0, 0). Similarly the plane represented by the equation in the first row crosses the 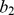 axis at 2 so a second point on the plane is (0, 2, 0). Finally, the plane crosses the 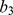 axis at 4 so that another point on the plane is (0, 0, 4). These three points determine the plane represented by the first equation in this three space. In the same manner we can determine the plane for the second row equation by finding where it crosses the three axes (2.50, 0, 0) (0, 1.667, 0), and (0, 0, 2.50); and for the third row equation (6, 0, 0), (0, 6, 0), and (0, 0, 3). Since two of these planes are not linearly dependent, they intersect one another and intersection will determine a line. On this line, the solution to the equations must lie. In (8) the third plane is not linearly dependent on the first two planes, so it will intersect this line at a point, and this point will determine the unique solution for this three equation system. This point of intersection (2.333, −1.667. 2.667) will be the same as the solution using algebraic means. A careful geometer would be able to generate this solution using the intersections of planes. Of course, we are interested in the visualization/intuition supplied by the geometric perspective and would not recommend such geometric constructions as a means for computing these results. For now, we simply need to visualize two planes intersecting in a line in a three space (imagine the three-space as a room) and another plane crossing that line. That point of intersection supplies the unique coordinates in a three-space and thus a unique solution for the parameter estimates.

Below (9), we depict a linear dependency where the third row equation is one-half the first row equation plus one-half the second row equation:
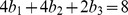


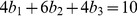
(9)

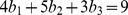
There is not a unique solution to this set of equations. If we constructed planes for two of these three equations they would intersect in a line, since any two of these equations do not form a linearly dependent set. This line will lie on the remaining plane, so that any solution on this line will be a solution to this set of equations. In order for there to be a unique solution, the remaining plane would have had to intersect the line formed by the intersection of the other two planes at a point.

A line in a space of three or more dimensions is typically described by using the vector equation for a line. This equation tells where all of the points on the line are in terms of the coordinates on each of the dimensions. For the first two equations in (9) the line of their intersection can be described by the following vector equation for the line:
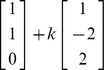
The intersection of the second two planes can be described by the same line as can the intersection of the first and third planes.

As noted, the remaining plane (the one not involved in the intersection) does not help us find a unique solution, since it does not intersect with the line of solutions at a single point: all of the points on the line lie on the remaining plane and thus there is no unique intersection point for the line and the plane. This makes sense because we could have used the first and third equation or the second and third equation and the line created by these intersections would be the same one. That is, all of the planes have this line lying on their surfaces. If the plane of the remaining equation were not linearly dependent on the other two equations, its plane would intersect the line established by the first two planes. (In constrained regression, we force the remaining plane to change direction and thus provide a unique solution under the constraint).

The null vector for (9) is (1, −2, 2)′ since:
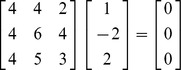
The null vector is parallel to the line established by the two intersecting planes. We label the line of intersection as the “line of solutions,” since any point on that line solves the set of equations that are rank deficient by one. Of course, these solutions are not the unique solutions that one obtains with linearly independent equations.

With three normal equations there is one more possibility in terms of linear dependency. The matrix of independent variables may be rank deficient by 2: that is, there may *not be* a set of two of these three equations that are linearly independent. There may be two linearly independent null vectors. This happens with the normal equations in (10) for which I have deliberately selected data to produce an 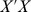
matrix that is rank deficient by two:
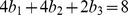


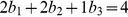
(10)

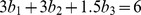
None of these planes intersect: all three of them coincide with one another. They fall in the same two dimensional subspace. For example, all three of these planes intersect the 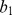 axis at (2, 0, 0); when 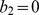 and 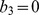 then 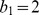 for all three of the equations. Similarly, for all three equations the plane intersects the 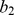 axis at (0, 2, 0) and the 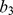 axis at (0, 0, 4). Clearly these three planes coincide. The solutions to these equations can reside anywhere on this “plane of solutions.” We can write these potential solutions as any one of the solutions (points on this plane) plus a scalar (*k*) times one of the directions of this plane plus a scalar (*s*) times the other direction of this plane. For example:
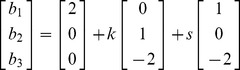
(11)If we set k  = 2 and s = –1, the point on this plane that results: (1, 2, −2). This works as a solution for (9) as does any point on this plane.

Not surprisingly the two vectors that are multiplied by *k* and *s* are the null vectors for (9), that is:
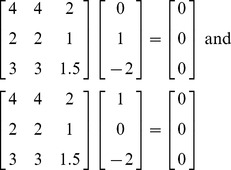
(12)These two null vectors are not linearly dependent on one another, and any other null vectors that produce the zero vectors are linearly dependent on these two null vectors. The null space in this case is a plane that passes through the origin (0, 0, 0) that can be described as:
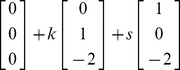
(13)The solutions to the equations lie on a plane of solutions and that plane is parallel to the null space which is a plane.

This methods section was written at the suggestion of a reviewer and designed to make the results that follow more intuitively understandable. To summarize: this paper examines the normal equations: 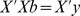. Any solution to these equations provides a least squares solution; even in situations where there are linear dependencies and, thus, an infinite number of solutions. Any one of the solutions provides a least squares solution. The problem with a linear dependency is not that we cannot find *a* solution; the problem is that there is not a unique solution.

*Without linearly dependent equations*, we find that in the two-variable situation the normal equations consist of two equations for lines and these lines intersect in the two-dimensional solution space and provide a unique solution to the equations. With three independent variables there are three normal equations and each one is the equation for a plane. These three planes intersect at a unique point in the three-dimensional solution space providing a unique solution to the equations. Venturing beyond these intuitive two- and three-dimensional cases, the generalization/extension is straightforward, but the terminology and visualizations are more difficult. With four independent variables there are four normal equations. Each represents a three-dimension hyper-plane (one up from a two dimensional plane with three independent variables). If there are no linear dependencies, these four three-dimensional hyperplanes intersect in a point in the four-dimensional solution space and provide a unique solution.

*With linear dependency* we saw that in the two variable case the two lines representing the two normal equations coincide (they lie on one another); they do not intersect and any solution on these coinciding lines, “the line of solutions,” solves the two normal equations. In the three independent variable situation where the three normal equations represent planes; if the matrix of independent variables is rank deficient by one (there is a set of two linearly independent equations), then two of the planes intersect in a line in the three-dimensional solution space. The remaining plane, however, does not intersect this line at a unique point, the line of solutions lies on the plane. If the matrix of independent variables is rank deficient by two: there are two linearly independent null vectors and all three planes coincide. Any point on this “plane of solutions” solves the normal equations. In a four space, when the matrix of independent variables is rank deficient by one, three of the three-dimensional hyperplanes intersect in a line (the line of solutions), but the remaining three dimensional hyperplane does not intersect the line of solutions at a unique point.

In our context, the null vector (*v*) is a vector that does not consist of all zeros and for which 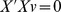. There is one such vector when a matrix is rank deficient by one, there are two such linearly independent vectors when the matrix is rank deficient by two and these two linearly independent vectors define a null space that is a plane. This null space is parallel to the plane of solutions. By extension there are *q* such vectors when the matrix is rank deficient by *q* and these *q* linearly independent null vectors form a null space that is a *q*-dimensional hyperplane. This *q*-dimensional hyperplane is parallel to the *q*-dimensional hyperplane of solutions.

These sorts of extensions provide the basis for our results section. Kendall [18] provides a more technical basis for some of these results, but he focuses on the full column rank situation. We include Appendix S1, based in part on [18], which helps to formalize these extensions. Readers may want to refer to Appendix S1 as they read the Results section. The algebra and the geometry, of course, are consistent. The results below necessarily repeat portions of the methods section.

## Results

### Identified Models: No Rank Deficiency

When a regression model with two independent variables is identified, there are two independent normal equations (for lines) in a two space, and the two lines intersect at a unique point providing a unique solution to the equations. In a three space with three independent normal equations (for planes), two of the planes intersect in a line and the remaining plane intersects the line at a unique point providing a unique solution to the equations. In a four space with four independent equations (for three-dimensional hyperplanes), two of the hyperplanes intersect in a plane, a third hyperplane intersects the plane in a line, and the fourth hyperplane intersects the line at a unique point providing a unique solution to the equations. In an *m*-space with *m* independent equations (each equation represents an (*m*−1)-dimensional hyperplane), the *m* hyperplanes intersect at a unique point providing a unique solution to the equations.

When the matrix of independent variables is of full column rank (there is no rank deficiency), finding unique solutions for each of the independent variables is straightforward using a regular regression program or matrix algebra. We could constrain one or more of the regression coefficients, if we choose; but if we did, we would likely degrade the fit of the model by changing the orientation of one or more of the hyperplanes so that their intersection is at a different point than the identified solution. This would create a solution that was not a least squares solution. One could do this to see if the constraint significantly degraded the fit of the model. Our focus in this paper, however, is on the geometry of rank deficient models in which the constraints are used to provide *a solution* to the models; *models that with the constraint are just identified*.

### Rank Deficient by One Models

One less than full column rank is the situation illustrated in each of the empirical examples cited in the introduction. In the case with three independent variables with a rank of two, we can determine the line on which the solutions must fall (two of the normal equations intersect in a line): we label this *the line of solutions*; but the remaining plane (equation) does not intersect this line (the line of solutions lies on this plane). We can determine the line on which the solution must fall, but not the point on that line. The constrained regression solution to this dilemma is to set the direction of the plane so that it intersects the line on which the solution must fall. One way to do this is to use a generalized inverse based on a particular constraint [4]. This provides *a solution* to the system of equations (under that constraint). One can use any appropriate generalized inverse without worrying about the constraint it imposes, but it most certainly imposes a constraint.

To make our discussion more concrete, we present an example with three equations in which the rank of the matrix is two. We have centered all of the variables in this analysis by subtracting their mean values from each of the values of the observations on these variables. Our reason for doing so is to allow us to visualize the solutions with three independent variables in a three-space. Alternatively, we could have included a column of ones in the *X*-matrix for the intercept and used just two independent variables in our example.

We use the normal equations for 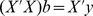 below for this example:
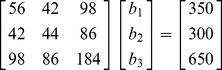
(14)The linear dependency is evident in 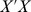 matrix. The null vector (the vector that when multiplied times
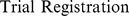
produces the zero vector) is (1, 1, –1). This representation of the null vector is unique up to multiplication by a scalar.

**Figure 2:**
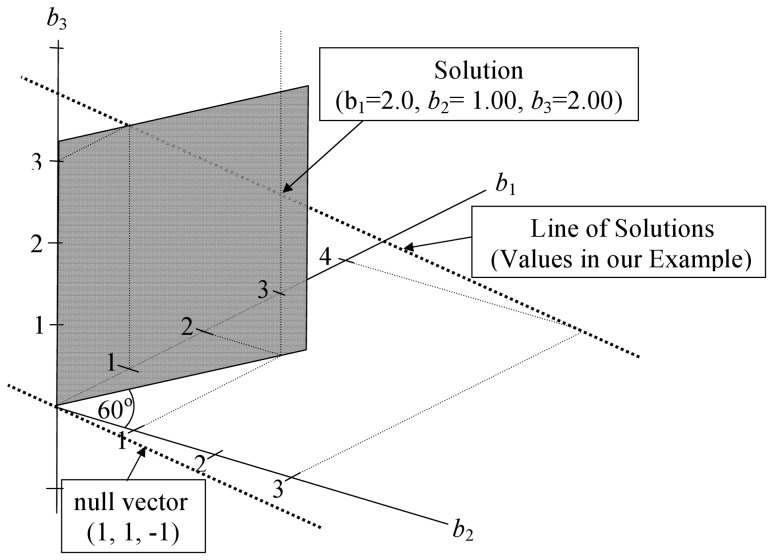
Geometric view of constrained regression in three-dimensions with one linear dependency. The constraint is .5*b*_1_ =  *b*_2_. The null vector intersects the origin, the line of solutions (on which the solutions must fall) is parallel to the null vector. The constrained solution plane intersects the line of solutions at (2.0, 1.0, 2.0).

Figure 2 presents this problem in a three-space in which the axes represent the unknown regression coefficients. We can represent the null vector in the three-space created by the axes for *b*_1_, *b*_2_, and *b*_3_ as a line extending through the points (1, 1, –1) and (0, 0, 0). It is represented by the left most darkly stippled line. The right most darkly stippled line is labeled the line of solutions – it is the line on which the solutions to the constrained regression must fall. For the data in (14) the line of solutions crosses the *b*_1_–*b*_2_ plane at (4, 3, 0), because when 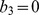, 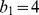 and 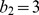 provide the correct solution for all three equations. Similarly, the line of solutions crosses the *b*_1_–*b*_3_ plane at (1, 0, 3). We can describe this line using the vector equation for a line 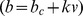 by choosing any one of these points as a solution 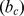 and adding *k* times the null vector (*v*) to it: (4, 3, 0)′ + *k*(1, 1, −1)′. This guarantees that the line of solutions and the null vector are parallel (they share the same direction). The line of solutions also represents the intersection of two of the planes described by the normal equations in (14). The remaining normal equation (plane) does not intersect the line of solutions: the line of solutions lies on it. The question is which solution on the line of solutions we will choose? We can choose it explicitly using constrained regression or implicitly using any generalized inverse.

A solution is achieved by constraining the direction of the remaining plane; *in general* the constrained solution plane will intersect the line of solutions at a point which provides *a* solution to the equations. We use the term *in general*, because if the plane is constrained to be in the direction of the line of solutions, it will not intersect the line. For example, setting the constraint *b*_1_ =  *b*_2_ for this data will not change the orientation of the plane and will not produce *a* solution. This happens if we constrain b_1_ =  − *b*_3_; on the other hand, *b*_1_ =  − *b*_2_, *b*_1_ =  *b*_3_, or. 5*b*_1_ =  *b*_2_ will produce a solution, as will most other constraints. In Figure 2, we depict the constrained solution plane under the constraint. 5*b*_1_ =  *b*_2_. It has a slope of.5 with reference to the *b*_2_-*b*_1_ axis (an increase of 1 on *b*_1_ is associated with an increase of. 5 on *b*_2_). The constrained plane is shaded and intersects the line of solutions at (2, 1, 2). This is the solution under the constraint that. 5*b*_1_ =  *b*_2_. We have kept some “construction lines” in the figure that are light and stippled to highlight a few important points in the construction of the figure.

A careful geometer could find the solution to this constrained regression in this three dimensional case graphically. For this case, and others involving more dimension, Mazumdar, et al. [4] show how we can use generalized inverses that correspond to particular constraints. For example, the solution that corresponds to the Moore-Penrose inverse is (1.67, .67, 2.33). The Moore-Penrose corresponds to the constrained solution that is orthogonal to the null vector [(1.67, .67, 2.33)(1, 1, −1)′  = 0] and can be implemented using the system of Mazumdar, et al. [4], by using the constraint *b*_1_ =  *b*_3_– *b*_2_ in a constrained regression program, or by using the Moore-Penrose inverse. Proceeding graphically, the constrained plane would be orthogonal to the null vector (1, 1, −1) and intersect the line of solutions at (1.67, .67, 2.33).

It is, of course, more difficult to draw a figure for the situation in which the rank deficiency is one and there are four independent variables. In this case there are four equations representing four three-dimensional hyperplanes. The line of solutions is determined by the intersection of three of these hyperplanes and when we find one of the solutions to the normal equations we can write the line of solutions as 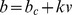. The line of solutions is parallel to the null vector. Unfortunately, the line of solutions does not intersect the remaining hyperplane.

As an example, we use the following normal equations 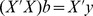:
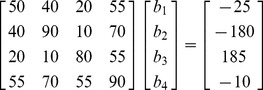
(15)


For (15) the null vector is (1, 1, 1, −2). When the constraint is *b*_1_ =  –*b*_3_, the solution vector is (−1, −4, 1, 3)′. This solution vector certainly works in (15); for example –1×50–4×40+1×20+3×55 = –25 for the first row, and similarly for the remaining rows of (15). Thus, the line of solutions may be written as (−1, −4,1,3)′ + *k*(1,1,1, −2)′. This completely specifies the line of solutions; and we perhaps can imagine such a line in a four dimensional space.

It is more difficult to imagine the remaining three-dimensional hyperplane. It is the orientation of this hyperplane that is constrained in four dimensional space to obtain a solution to the equations. With the linear dependency this three-dimensional hyperplane, represented by one of the normal equations, does not intersect the line of solutions (determined by the other three hyperplanes). We must use a constraint to force this hyperplane to cross the line of solutions at a unique point. In this case, if we use the constraint is *b*_1_ =  –*b*_3_, the constrained hyperplane has as slope of −1 on the *b*_1_–*b*_3_ plane: an increase of one on *b*_1_ is associated with a decrease of 1 on *b*_2_ (note, the hyperplane must cross the (0, 0, 0, 0) point in the four-dimensional solution space). This change in orientation constrains this hyperplane to cross the line of solutions at a unique point. Again we might set a constraint that yields a hyperplane that does not intersect the line of solutions. In this case, we might have set *b*_1_ =  *b*_3_, and the hyperplane will not intersect the line. In general, however, for almost all constraints the hyperplane will intersect with the line of solutions.

The extension to *m* dimensions is straightforward. There are *m* equations representing *m* (*m*–1)-dimensional hyperplanes. The line of solutions (a line in *m* space) is determined by the intersection of *m*–1 of these hyperplanes. Its vector equation for the line of solutions is 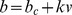; where *b*, *b_c_*, and *v* each have *m*-elements. The remaining hyperplane does not intersect the line of solutions. The single constraint that we place on the remaining hyperplane, in general, reorients it in the *m*-space, and results in the constrained hyperplane intersecting the line of solutions at a single point that yields *a* solution to the system of *m* equations.

### Rank Deficient by Two Models

When the *X*-matrix is two less than full column rank, it is still possible to visualize the solution in a three-dimensional space. To do so, we introduce a new set of normal equations 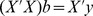:
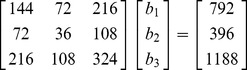
(16)The linear dependencies are evident in the 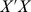 matrix. There are two linearly independent null vectors (1, −2, 0) and (1, 1, −1). These two vectors define the null space, which in this case is a plane (a hyperplane of two-dimension: a plane).

Once we solve for one of the infinity of possible constrained solutions, it is a simple matter to write the plane of solutions using the vector equation for a plane. The solution must lie on the plane defined as: 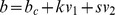 where 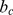 is any particular constrained solutions, 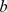 represents all of the possible solutions, *k* and *s* are scalars, and *v*_1_ and *v*_2_ are two linearly independent null vectors. (There are other ways to represent these two linearly independent null vectors, but all other ways are linearly dependent on these two null vectors.) In this situation, all three planes determined by the three normal equations coincide with each other and form the plane of solutions. Two constraints are required to determine a solution in this rank deficient by two case. We can view one of the constraints as shifting the orientation of one of the two planes so that it intersects (in general) with one of the other two planes producing a line under the first constraint. The second constraint orients this line so that it intersects (in general) with the plane of solutions. Note the terminology as we move from the rank deficient by one to the rank deficient by two situation. *The line of solutions* from the previous section is now *the plane of solutions.* The plane of solutions is the subspace (two-dimensional) parallel to the null space on which the solution must lie.

For the data in (16) a solution to the normal equations is (5.5,0,0), so we can define the plane of solutions as (5.5,0,0)′ + *k*(1, −2,0)′ + *s*(1,1, −1)′. All of the solutions using linear constraints will fall on this plane: the question is where. The answer when using constrained regression depends upon the constraints that we place on the solution.

**Figure 3:**
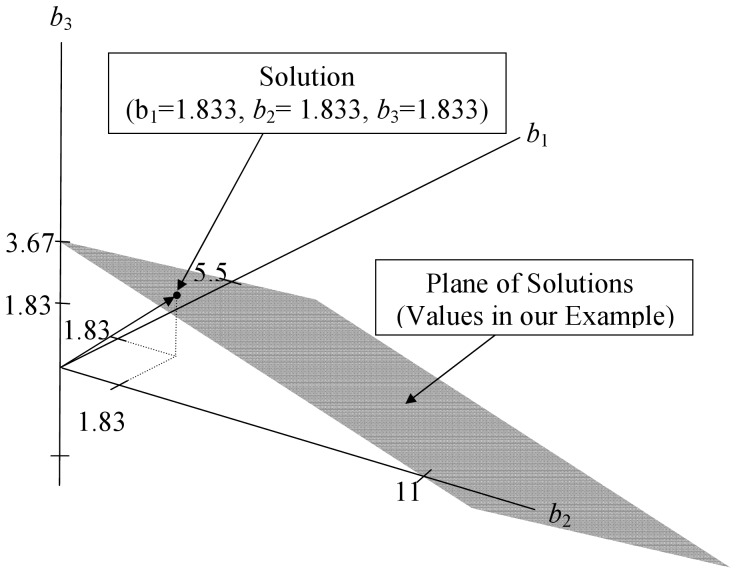
Geometric view of constrained regression in three dimensions with two linear dependencies. The constraints are *b*_1_ =  *b*_2_ and *b*_2_ =  *b*_3_. The plane of solutions (on which the solutions must fall) is parallel to the null space (not shown). The constrained solution line intersects the plane of solutions at (1.833, 1.833, 1.833).

In Figure 3, to avoid “cluttering,” we have not depicted the null space (a plane that is parallel to the plane of solutions and passes through (0,0,0)). The plane of solutions is depicted in Figure 3 and passes through the points (5.5,0,0), (0,11,0), and (0,0,3.67). All of these points fall on the plane of solutions, which can be verified using the vector equation for this plane. Since the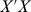 is two less than full column rank, we must set two constraints on the solution. In Figure 3, we use the constraints *b*_1_ =  *b*_2_ and *b*_2_ =  *b*_3_. Together they constrain the solution to lie on a line that is equiangular (forming 45 degree angles) with each of the axes. The solution using these two constraints is (1.833, 1.833, 1.833), which is depicted in Figure 3 as where the arrow from (0,0,0) intersects the plane of solutions. It is easy to show that this solution works for the data in (16). It provides a least squares solution, but so do an infinite number of other solutions based on different combinations of two constraints that force a line to intersect the plane of solutions.

In a four-space with 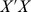 rank deficient by two there are two linearly independent null vectors and the null space is a plane. Each of the four normal equations represents a three-dimensional hyperplane and two of them intersect to determine the plane of solutions, which is parallel to the null space. The two remaining three-dimensional hyperplanes are linearly dependent on the two hyperplanes that intersected to form the plane of solutions. Placing a constraint on one of the two remaining hyperplanes will, in general, lead to its intersection with the other remaining hyperplane and determine a plane (a two-dimensional hyperplane). This plane does not intersect the plane of solutions. The second constraint will determine the direction of this plane that, in general, will intersect the plane of solutions at a single point. Here, the geometry strains our intuition, but two planes in a four space, in general, intersect in a point [18], [19]. Fortunately, both the null space and the solution space being planes are reasonably intuitive even if they are embedded in a four space.

When we increase the number of dimensions, the solutions follow this same geometric pattern. Each of the *m* normal equations represents an (*m*–1)-dimensional hyperplane. As long as there are just two linearly independent null vectors, there will be a plane of solutions: 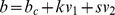. This plane of solutions is determined by the intersection of *m*–2 of the hyperplanes (all but two of the hyperplanes). The two remaining hyperplanes are linearly dependent on the *m*–2 hyperplanes that intersected with each other. We need to constrain these (*m*–1)-dimensional hyperplanes so that they intersects with each other. The intersection results in an (*m*–2)-dimensional hyperplane and the second constraint is used to constrain the direction of this hyperplane. In general, this constrained (*m*–2)-dimensional hyperplane and the two-dimensional plane of solutions will intersect at a point in the *m*-dimensional solution space and thus will provide a unique solution to the system of equations under the constraints imposed. (Again, the reader is referred to Appendix S1 for some rules for the intersection of hyperplanes for cases described in this paper.).

### The General Case

In the general case, if we have an *m* column matrix of independent variables, there are *m* normal equations (one for each row). Each equation represents an (*m*–1)-dimensional hyperplane. If the *m* column matrix is rank deficient by *d*, then the null space is of *d*-dimensions and the hyperplane of solutions is *d*-dimensional. The hyperplane of solution can be represented by 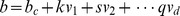. This *d*-dimensional hyperplane of solutions is determined by the intersection of *m*–*d* of the hyperplanes. To solve the system of equations, we need *d* constraints. We use *d*–1 of these constraints to produce an intersection between the *d* remaining hyperplanes. These intersections result in (*m* – *d*)-dimensional hyperplane. The final constraint orients this (*m* – *d*)-dimensional hyperplane. These two hyperplanes (the *d*-dimensional hyperplane of solutions and the constrained (*m* – *d*)-dimensional hyperplane), in general, intersect in the *m* – dimensional solution space at a unique point. Thus, they provide a unique solution to the system of equations under the constraints imposed.

## Discussion

We have examined setting specific constraints to find a solution to a system of normal equations when the matrix of independent variables is less than full column rank. Our emphasis has been on the rows of the normal equations; each row representing an (*m* –1)-dimensional hyperplane. We have used the null vectors to help visualize the hyperplane of solutions that is of the same dimension as the null space and is parallel to it. The *d*-dimensional hyperplane of solutions is created by the intersection of *m* – *d* of the (*m* –1)-dimensional hyperplanes represented by each of the rows of the normal equations. Although there are an infinite number of solutions to the normal equations – we know that they lie in this space. By appropriately constraining the orientation of the *d* remaining (*m –*1)-dimensional hyperplanes, we can produce a solution to the normal equations *that is unique given the constraints*.

Computationally, we can find these constrained solutions by creating a generalized inverse based on the constraint [4]. It is important to note that even when we do not deliberately produce a generalized inverse with a particular constraint, any generalized inverse produces a constrained solution. In this sense, the geometry of using generalized inverses to solve these normal equations that are rank deficient is the same as when using constrained regression. Our discussion has focused on the geometric interpretation of constrained regression from the row perspective by focusing on the rows of the normal equation and their intersections. In some ways this perspective may be more difficult than the column perspective when the number of dimensions is large [20], but there are geometric intuitions/insights to be gained by taking this row perspective.

It is especially intuitive to think of the line of solutions and the plane of solutions in rank deficient by one and by two situations. The row geometry emphasizes that the unconstrained intersections among the row equations provide, to a large extent, what we know about the solution – it must fall on this space: a space that is parallel to the null space. It is helpful to think of the constraints as arranging the remaining hyperplanes in such a way that they all intersect with each other (if there is more than one). The hyperplane created from these constrained intersections (when there is more than one “remaining” hyperplane) is then oriented in such a way as to intersect with the hyperplane of solutions. This intersection produces a solution to the normal equations under the constraints applied. These are very helpful insights into how generalized-inverses/constrained-regression work.

How can this geometry be applied to a particular problem to help us gain insight into what is “going on” in the analysis? Using the Age-Period-Cohort model as an example of a rank deficient model which often is “solved” using constrained-regression/generalized-inverses to produce a least squares solution. The geometry lays out what the problem in this rank deficient by one case. A set of all of the independent variables but one are linearly independent. The intersection of the normal equations, for all but one of the normal equations, forms a line: a line of solutions. The remaining normal equation can be represented by a hyperplane, but this hyperplane does not intersect the line of solutions at a point. Constrained-regression/generalized-inverses change the orientation of this hyperplane so that it intersects the line of solutions and provides one of the solutions on the line of solutions. That solution is a least squares solution. Sometimes a constraint that we impose in constrained regression does not “work” in terms of providing a solution. This can occur because the constraint does not change the direction of the linearly dependent hyperplane and so it does not intersect the line of solutions. It is important to remember that this solution depends on the constraint, and we would recommend that anyone using such a constraint do so on the basis of theory/substantive considerations. The line of solutions is what we know from the data. We can determine this line from the data – it is identified. Although we do not consider it in this paper, this line can be used to derive other identified characteristics for the Age-Period-Cohort model [21], the so called “estimable functions” [22]–[24].

## Supporting Information

Appendix S1 **Six helpful points for describing the intersection of hyperplanes.**
(DOC)
