# Supplementary material for: Visualizing Rank Deficient Models: A Row Equation Geometry of Rank Deficient Matrices and Constrained-Regression
Source: PLoS One. 2012 Jun 19;7(6):e38923. doi: 10.1371/journal.pone.0038923 (PMC3378569; doi:10.1371/journal.pone.0038923)
Supplement: Appendix S1 — Six helpful points for describing the intersection of hyperplanes. (DOC) [file pone.0038923.s001.doc]

## Appendix S1

This paper deals with *m* independent variables and *m* normal equations (for ease of presentation, we assumed that the variables are in deviations score form).

Below we present six points that may be helpful in establishing the hyperplanes formed by intersection with other hyperplanes in the *m* – space. The first five points concern situations in which all of the hyperplanes intersect “naturally,” the situation with no linear dependencies.

**1)** There are *m* hyperplanes (representing normal equations) in an *m*-dimensional solution space.

**2)** Each of the normal equations represents an (*m* – 1)-dimensional hyperplane.

**3)** When hyperplanes representing two linearly independent (*m* – 1)-dimensional hyperplanes intersect, the intersection is an (*m* – 2)-dimensional hyperplane. (Think of a three-dimensional space where each of the normal equations represents a plane [a (3 – 1)-dimensional hyperplane] and the intersection of these two planes is a line [a (3 – 2)-dimensional hyperplane]).

**4)** The intersection of an (*m* – *p*)-dimensional hyperplane with an (*m* – *s*)-dimensional hyperplane is an (*m* – (*p* + *s*))-dimensional hyperplane. (In a four-space the intersection of two planes [(4 – 2)-dimensional hyperplanes] is a point [an (*m* – (2+2)) or (4 – 4)-dimensional hyperplane]).

**5)** In an *m* dimensional solution space, when *p* + *s* = *m* the hyperplanes (if they intersect) intersect in a point. (For example, an (*m* – *s*)-dimensional hyperplane, in general, intersects an (*m* – *p*)-dimensional hyperplane when *p* + *s* = *m*.)

Our problem with linearly dependent equations is that not all of the hyperplanes intersect with one another unless they are constrained to intersect.

**6)** In the linear deficient by *q* case *m* – *q* of the hyperplanes intersect creating a hyperplane that is of *m* - (*m* – *q*) dimensions. That is, the hyperplane created by the intersections is of *q*-dimensions and this is of the same dimensions as the null space. In the rank deficient case we constrain the remaining *q* hyperplanes to intersect each other and to intersect the solution space. They intersect with each other in an (*m* – *q*)-dimensional hyperplane. Using (5) above, when these two hyperplanes are constrained to intersect with each other the result is a [*m* – ((*m*–*q*)+*q*)]-dimensional hyperplane. That is, a 0-dimensional hyperplane: a point. This is one way to view how constrained regression works geometrically.
